# Could the Microbial Profiling of Normal Pancreatic Tissue from Healthy Organ Donors Contribute to Understanding the Intratumoral Microbiota Signature in Pancreatic Ductal Adenocarcinoma?

**DOI:** 10.3390/microorganisms13020452

**Published:** 2025-02-19

**Authors:** Francesca Tavano, Alessandro Napoli, Domenica Gioffreda, Orazio Palmieri, Tiziana Latiano, Matteo Tardio, Fabio Francesco di Mola, Tommaso Grottola, Markus W. Büchler, Marco Gentile, Anna Latiano, Tommaso Mazza, Francesco Perri

**Affiliations:** 1 Division of Gastroenterology and Endoscopy, Fondazione IRCCS “Casa Sollievo della Sofferenza” Hospital, Viale Cappuccini 1, 71013 San Giovanni Rotondo, FG, Italy; 2 Bioinformatics Laboratory, Fondazione IRCCS “Casa Sollievo della Sofferenza” Hospital, Viale Cappuccini 1, 71013 San Giovanni Rotondo, FG, Italy; 3 Department of Surgery, Fondazione IRCCS “Casa Sollievo della Sofferenza” Hospital, Viale Cappuccini 1, 71013 San Giovanni Rotondo, FG, Italy; 4 Unit of Surgical Oncology, Casa di Cura Pierangeli, 65124 Pescara, PE, Italy; 5 Department of Medical, Oral and Biotechnological Sciences, University “G. d’Annunzio” Chieti-Pescara, 66100 Chieti, CH, Italy; 6 Department of Innovative Technologies in Clinical Medicine and Dentistry, University “G. d’Annunzio” Chieti-Pescara, 66100 Chieti, CH, Italy; 7 Botton-Champalimaud Pancreatic Cancer Center, Champalimaud Foundation, 1400-038 Lisbon, Portugal; 8 Department of General, Visceral and Transplantation Surgery, Heidelberg University Hospital, 69120 Heidelberg, Germany

**Keywords:** pancreatic ductal adenocarcinoma, microbiota, intratumoral bacteria, tumor microenvironment, 16S rRNA gene sequencing, carcinogenesis

## Abstract

Pancreatic ductal adenocarcinoma (PDAC) is associated with intratumoral microbiota changes. However, defining the normal pancreatic microbial composition remains a challenge. Herein, we tested the hypothesis that the microbial profiling of normal pancreatic tissue from healthy organ donors (HC) could help in determining the signature of microbiota in PDAC. Matched pairs of tumor and normal tissues from PDAC patients (n = 32) and normal pancreatic tissues from HC (n = 17) were analyzed by 16S rRNA gene sequencing. Dissimilarities in all the beta metrics emerged in both normal samples and tumor samples, compared to HC (Bray–Curtis dissimilarity and Jaccard distance: *p* = 0.002; weighted UniFrac distances: *p* = 0.42 and *p* = 0.012, respectively; unweighted UniFrac distance: *p* = 0.009); a trend toward a lower Faith’s phylogenetic distance was found at the tumor level vs. HC (*p* = 0.08). Within PDAC, a lower Faith’s phylogenetic distance (*p* = 0.003) and a significant unweighted UniFrac distance (*p* = 0.024) were observed in tumor samples vs. normal samples. We noted the presence of a decreased abundance of bacteria with potential beneficial effects (*Jeotgalicoccus*) and anticancer activity (*Acinetobacter_guillouiae*) in PDAC vs. HC; bacteria involved in immune homeostasis and suppression of tumor progression (*Streptococcus_salivarius*, *Sphingomonas*) were reduced, and those implicated in tumor initiation and development (*Methylobacterium-Methylorubrum*, *g_Delftia*) were enhanced in tumor samples vs. normal samples. Metagenomic functions involved in fatty acid synthesis were reduced in normal samples compared to HC, while peptidoglycan biosynthesis IV and L-rhamnose degradation were more abundant in tumor samples vs. normal samples. Future prospective studies on larger populations, also including patients in advanced tumor stages and considering all potential existing confounding factors, as well as further functional investigations, are needed to prove the role of microbiota-mediated pathogenicity in PDAC.

## 1. Introduction

Pancreatic ductal adenocarcinoma (PDAC), the most common form of pancreatic cancer, represents the fourth-leading cause of death from cancer, with a 5-year survival rate of 12%, and it has been estimated that it will become the second-leading cause of cancer-related deaths in the Western world in 2030 [1].

In the past several decades, several efforts have been made toward trying to understand how PDAC develops and progresses while acquiring such aggressive features. PDAC is characterized by the presence of strong homogeneity in key driver mutations, and by differences in the tumor cell-intrinsic transcriptional and epigenetic profiles [2]. Furthermore, it is now recognized that the microbiota contributes significantly to the heterogeneity of tumor phenotypes. The evidence shows that PDAC is associated with unique oral, gut, and pancreatic microbiota signatures [3].

The concept of intratumoral microbiota, i.e., the microorganisms present within the tumor, arose from evidence arising across several cancers suggesting that bacteria could be detected within the tumor microenvironment [4]. As for the pancreas, although this organ was long considered sterile, a number of studies have established that it may be colonized by bacteria via different routes: microorganisms originating in the upper gastrointestinal tract could enter the pancreas by reflux into the pancreatic duct and then penetrate into the pancreatic parenchyma through the large/little papillae, while microbiota situated further away, such as in the colon, may migrate to the pancreas through the lymphatic system; under pathological conditions, such as inflammation-driven damage to the intestinal barrier and increased intestinal permeability, bacteria could also translocate to the pancreas along with the blood circulation [5]. The microbiota can influence the composition of the stroma and of the immune cells and can stimulate persistent inflammation, causing alterations in the antitumor immune system, and leading to changes in cellular metabolism in the tumor microenvironment [6,7]. Dissecting the causality between intratumoral microbiota and the PDAC development and progression, and elucidating the relationship between intratumoral microbes and host tumors could contribute to the detection of microbial composition as a biomarker usable in clinical settings for patients with PDAC.

Several authors analyzed the microbiome of the pancreas via 16S ribosomal RNA gene sequencing, with some studies reporting a different bacterial composition in PDAC compared to the normal pancreas, and others highlighting a similar bacterial community in the pancreas between normal and disease states [8,9,10,11]. Furthermore, potential effects of bacteria on PDAC prognosis, survival, and gemcitabine treatment outcomes have also been uncovered [9,10,11,12,13]. However, the studies reveal a lack of consistency and a variability in the definition of “normal” (i.e., the normal pancreas consisted of specimens from a non-cancerous pancreas, from a non-malignant surgical margin from a malignant surgical specimen, or from organ donors), and sometimes this was not clearly defined.

To our knowledge, up to today, no study has concomitantly characterized the microbiota in matched cancer and normal pancreatic tissues from patients who have undergone pancreatic resection for PDAC, as compared to normal pancreatic tissue obtained from a healthy organ-donor pancreas. Herein, we assume that such an approach could help determine the intratumoral microbiota signature in PDAC net of the microbiota from non-cancer-afflicted subjects. The hypothesis was that the microbial profiling of normal pancreatic tissue from healthy organ donors could be a critical step towards defining the intratumoral microbiota signature in PDAC, and aid in a better understanding of the contribution of bacteria in pancreatic carcinogenesis.

## 2. Materials and Methods

### 2.1. Clinical Specimens

Tissue specimens from 32 patients undergoing pancreatic resection for pancreatic cancer were collected and retrospectively assessed at Fondazione “Casa Sollievo della Sofferenza” IRCCS Hospital, San Giovanni Rotondo, Italy. Five out of the thirty-two patients underwent pre-operative endoscopic procedures: endoscopic ultrasound-guided fine-needle aspiration (EUS-FNA) was performed in two patients; and endoscopic retrograde cholangiopancreatography (ERCP) was carried out in two patients; while in one other patient, both procedures were used. The final diagnosis of PDAC was established by histologic examinations, and the coexistence of chronic pancreatitis was ruled out. Resected PDAC (T-PDAC) and adjacent normal tissues (N-PDAC) were taken separately. The control group consisted of normal human pancreatic tissue samples obtained from 17 healthy non-cancer-afflicted individuals (N-HC) through an organ donor program at the University of Heidelberg in Germany. The research was carried out in accordance with the Helsinki Declaration, and was approved by the hospital’s Ethics Committee (Prot. N.69 CE/2021, and No. 301/01). All subjects provided signed informed consent forms.

The tissue specimens were flash-frozen in liquid nitrogen and stored pending DNA extraction. Demographic information, along with information about clinical presentation and anamnestic information (i.e., presence of obstructive juice, diabetes, cancer antigen 19-9 (CA 19-9) serum marker levels, and personal and family history of cancer), as well as clinical pathological features associated with PDAC, were collected from the cases in the group. The data are shown in Table 1. No information as to dietary habits or drug treatment with antibiotics was available from the cases and controls enrolled in the study. The genders and ages of the controls were also unknown.

### 2.2. DNA Extraction and Processing

The gentleMACS Dissociator (Miltenyi Biotec, Bergisch-Gladbach, Germany) was utilized for the process of the homogenization of the tissue samples, and total DNA was extracted using the AllPrep Power viral DNA/RNA Kit (Qiagen, Hilden, Germany), in accordance with the manufacturer’s guidelines. Briefly, each frozen tissue sample was placed into a gentleMACS M Tube containing 750 μL pre-heated Solution PM1 Solution (provided by the extraction kit) supplemented by 150 μL of Phenol/CIA. The M tube was tightly closed, turned upside-down in one quick move, and attached upside-down onto the sleeve of the gentleMACS Dissociator. GentleMACS Program RNA_02 for frozen tissue was run. After termination of the program, the M Tube was detached from the gentleMACS Dissociator, and 750 μL of the homogenized sample was transferred to the Power Bead Tube provided with the extraction kit to proceed with DNA isolation according to the manufacturer’s recommendations. DNA quantity was examined using a NanoDrop ND-1000 spectrophotometer (Thermo Fisher Scientific, Inc., Somerset, NJ, USA).

The microbial composition was studied by sequencing the amplified V3 to V4 hypervariable region of the 16S rRNA gene according to the Illumina 16S Metagenomic Sequencing Library preparation guide (Par#15044223 Rev.B) [14], with some modifications in the amplicon PCR step concerning the use of the Taq Phusion High-Fidelity (Thermo Fisher Scientific, Sunnyvale, CA, USA), and the set-up of two separate PCR reactions. The first PCR reaction was run in the presence of the following pair of primers: GM3F: 5′-AGAGTTTGATCMTGGC-3′)/907R: 5′-CCGTCAATTCMTTTGAGTTT-3′; the amplicons from the first PCR were subjected to a second run of PCR using the following pair of primers: 341F: 5′-CCTACGGGNGGCWGCAG-3′/805R: 5′-GACTACHVGGGTATCTAATCC-3′ [15]. Briefly, the first PCR was carried out in 25 µL reaction volumes containing 2 µL of bacterial template DNA; the annealing temperature for primer pair 1 was set at 59 °C, and each reaction was run for 35 PCR cycles. Subsequently, 1 µL of the product of the first amplification reaction was used as the template for the second PCR reaction, which was performed in 25 µL volumes by using primer pair 2 with the 5′ addition of Illumina adapter overhang nucleotide sequences; the annealing temperature was set at 55 °C for 25 cycles of amplification. A no-template PCR negative control and a PCR positive control were added to each PCR run. The latter consisted of a bacterial template DNA from colon cancer tissue specimens, such as those previously used for the amplicon PCR step, and was performed directly with pair 2 according to the Illumina protocol [16]. Amplicons were identified through 3% agarose gel electrophoresis.

All the other steps of the protocol, including PCR Clean-Up, Index PCR, PCR Clean-up 2, Library Quantification, Normalization, and Pooling Library Denaturing and MiSeq Sample Loading, were carried out following the aforementioned preparation guide.

### 2.3. Microbiota Pipeline Analysis

The FastQC application was mainly used to check the quality of sequencing output; thereafter, sample reads were demultiplexed and analyzed with the QIIME2 v2024.2.0 suite [17]. The preliminary actions, which involved quality-checking of reads and filtering, de-replication, identification of chimeric reads, joining of paired-end reads, and determining clustering sequences, were executed utilizing the DADA2 [18] plugin, resulting in the creation of a raw feature table. The SILVA database (version 138) was used for taxonomic classification, employing a pre-trained Naive Bayes classifier downloaded from the QIIME 2 Data Resources 99% OTU (available at https://doi.org/10.1093/nar/gks1219, accessed on 28 October 2024). The completeness of the sampling of the microbial communities was assessed using rarefaction curve analysis with the QIIME2 diversity plugin. We eliminated contaminant sequences (i.e., those of mitochondrial/chloroplast origin) and filtered out features present in fewer than 5 samples or with a total frequency of less than 10. We removed contaminant sequences (i.e., of mitochondrial/chloroplast origin) and filtered out features occurring in fewer than 5 samples or with a total frequency below 10. Alpha- and beta-diversity metrics were calculated after rarefying the feature table to a depth of 4910 sequences per sample. Rarefaction curves were generated to ensure sufficient sequencing depth for downstream analyses. Phylogenetic diversity metrics were computed using a phylogenetic tree generated via the QIIME 2 phylogeny pipeline.

Subsequently, alpha diversity was assessed through Pielou’s evenness index, Shannon’s diversity index, the Simpson index, the number of observed OTUs, and Faith’s Phylogenetic Diversity [19,20,21,22,23], and beta dissimilarities were calculated through Bray–Curtis distance, Jaccard distance, weighted UniFrac, and unweighted UniFrac distances [24,25,26].

Principal Coordinates Analysis (“PCoA”) graphs were created utilizing the EMPeror application [27] for each beta-diversity metric to assess the heterogeneity of samples/experiments. Kruskal–Wallis and PERMANOVA statistical tests were utilized to identify variations in the aforementioned alpha indices and beta metrics across sample groups, respectively.

Variation in the taxa abundance profiles among groups was explored by compositional data analysis methods, i.e., QIIME2-ANCOM, Coda-lasso, Clr-lasso, and Selbal [28]. The input for QIIME2-ANCOM consisted of two filtered feature tables, collapsed, respectively, at the species and genus levels. Coda-lasso [29], Clr-lasso [30], and Selbal feature selection algorithms were additionally implemented (as described in https://malucalle.github.io/Microbiome-Variable-Selection/, accessed on 16 December 2024) in order to retrieve a sort of microbial signature in pairwise group comparisons (normal tissue from PDAC vs. healthy donors; tumor tissue from PDAC vs. healthy donors; and normal vs. tumor tissue from PDAC).

Functional metagenomic profiles were generated using the PICRUSt2 v2024.5 pipeline [31], which estimates the functional potential of microbial communities based on 16S rRNA gene sequencing data. Three datasets were analyzed: enzyme commission (EC) abundances, KEGG ortholog (KO) abundances, and pathway abundances. Picrust2 predicted counts for pathways (namely, the “unstratified pathway abundance”) via a count table, with pathways represented by their relative MetaCyc [32]. Differential abundance analysis was performed using the R package ALDEx2 v1.30.0. The analysis was conducted independently for EC, KO, and pathway abundance datasets. For each dataset, 128 Monte Carlo samples were generated (mc. samples = 128), and the Kruskal–Wallis test (test = “kw”) was used to identify significant differences in feature abundance between groups. The analysis yielded a set of raw results for each dataset, which were subsequently filtered to identify significant features. Features with adjusted *p*-values (kw.ep or glm.ep) less than or equal to 0.05 were retained for further interpretation. Statistical and graphical analyses were conducted in the R statistical environment, utilizing ggplot2 v3.5.1 [33] and Phyloseq v.1.42.0 [34].

### 2.4. Statistical Analysis

We conducted a correlation analysis to investigate potential associations between clinical features and microbial taxa. The OTU table was extracted from the BIOM file using the *load_table* function from the biom package in Python v3.10.4 and converted into a pandas DataFrame, utilizing the pandas library v1.5.3 for efficient data manipulation. Taxonomic annotations were joined to the OTU table and filtered to exclude entries with low confidence scores (<0.7) or uninformative taxonomy labels (e.g., “Unassigned” or generic levels such as d_Bacteria). The merged dataset, comprising clinical metadata and microbial abundance data, was used to compute Pearson correlation coefficients between microbial taxa and selected clinical features. Statistical significance was assessed by calculating *p*-values, which were adjusted using the Benjamini–Hochberg procedure to control the false discovery rate (FDR). Correlations with a coefficient > 0.7 and an adjusted *p*-value < 0.05 were considered significant.

## 3. Results

### 3.1. Study Design

Firstly, the microbial profile identified in N-HC was compared to microbes pointed out in T-PDAC and matched N-PDAC tissue specimens. The aim was to characterize the microbial diversity in pancreatic tissue specimens affected by PDAC compared to those unaffected by cancer and to define the most representative features and those variations in the microbial composition which would enable researchers to distinguish between these samples.

Secondly, the microbial compositions were compared in matched pairs of T-PDAC and N-PDAC tissue specimens. The intent was to determine the diversity and the composition of the microbial communities between different tissues within the PDAC cases and to recognize the features associated with the specific status of the pancreatic tissue (i.e., tumor/non-tumor) in these patients.

### 3.2. Characteristics of the Study Population

As shown in Table 1, 40.6% of the PDAC patients were less than 55 yrs old, and 65.6% were males. Obstructive jaundice and diabetes were present in 35.5% and 28.1% of patients, respectively. The concentration of the CA 19-9 tumor marker was about three times over the normal value of 37 U/mL in 48.3% of patients. Information about a personal or family history of cancer was available from all the patients enrolled in the study, with two patients having a prior history of cancer (6.3%) and three (9.4%) reporting a family history of cancer through their first-degree relatives. In over 90% of patients, the cancer was located in the pancreatic head, while a postoperative pathological examination indicated a mucinous PDAC in 18.8% of the cases. According to the American Joint Committee on Cancer (AJCC) tumor/node/metastasis (TNM) classification and staging system for PDAC [35], 13% of patients were in stage I, 34% in stage II, 50% in stage III, and 3% in stage IV. Neo-adjuvant chemotherapy was administered to 6.3% of patients three months before surgery and tissue sample collection, while 75.9% of patients were considered eligible for adjuvant chemotherapy after surgery.

### 3.3. 16S Amplicons Sequencing

After demultiplexing and quality control, the denoising of sequence data was performed with the DADA2-QIIME 2 algorithm. As shown in Supplementary Table S1 (Sheet S1), we obtained sample-specific sequencing yields ranging from 8573 to 59,742 good-quality reads.

Utilizing the raw feature table along with its related phylogenetic tree, we performed numerous rarefaction tests at various read-depth thresholds. This allowed us to determine the optimal sampling depth of 8573 reads with minimal sample loss (only one sample from N-PDAC tissue specimen), and a stable distribution of the alpha-diversity metrics across all the investigated groups.

### 3.4. Alpha and Beta Diversity of Microbial Communities

Alpha diversity was assessed by examining five metrics (Pielou’s evenness, Number of observed features, Shannon’s Entropy, the Simpson index, and Faith’s Phylogenetic Distance) and comparing group-specific distributions through Kruskal–Wallis tests, globally and pairwise. Table 2 presents the q-values for pairwise group comparisons, whereas boxplots illustrating diversity analyses are displayed in Supplementary Figure S1. When samples from N-PDAC and T-PDAC were compared to those from N-HC, only a trend toward a lower diversity in Faith’s Phylogenetic Distance index emerged in T-PDAC (q-value = 0.08), and no significant differences were found among all the other analyzed alpha index values (q-value > 0.05). Conversely, when comparing the microbiota between T-PDAC and N-PDAC tissue specimens, a statistically-significant lower diversity in the Faith’s Phylogenetic Distance index emerged at the tumor level (q-value = 0.003), while no significant differences were exhibited according to the other analyzed alpha indices (q-value > 0.05).

Beta diversity was evaluated by analyzing four metrics (Bray–Curtis, Jaccard, weighted UniFrac, and unweighted UniFrac) and comparing the beta dissimilarity metrics through the PERMANOVA pairwise test (Table 3); microbial community data were visualized through Emperor plots (Supplementary Figure S2). Comparison between N-PDAC and T-PDAC tissues and N-HC demonstrated a statistically significant difference regarding dissimilarity across all the examined indices (Bray–Curtis and Jaccard dissimilarity q-value = 0.002 at both non-tumor and tumor level; weighted UniFrac dissimilarity q-values = 0.042 and 0.012 at non-tumor and tumor levels, respectively; unweighted UniFrac dissimilarity q-value = 0.009 at normal and tumor levels). When we compared the microbiota between N-PDAC and T-PDAC, the differences among the samples were significant only for unweighted UniFrac dissimilarity (q-value = 0.024), while no significant differences were exhibited according to the other analyzed beta indices (q-value > 0.05).

**Figure 1:**
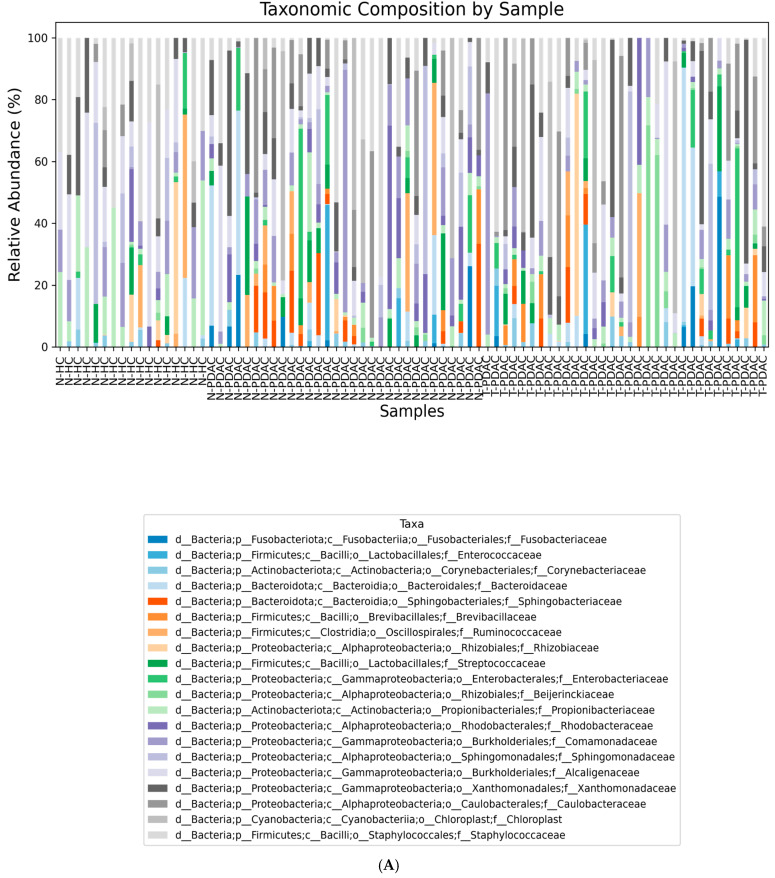

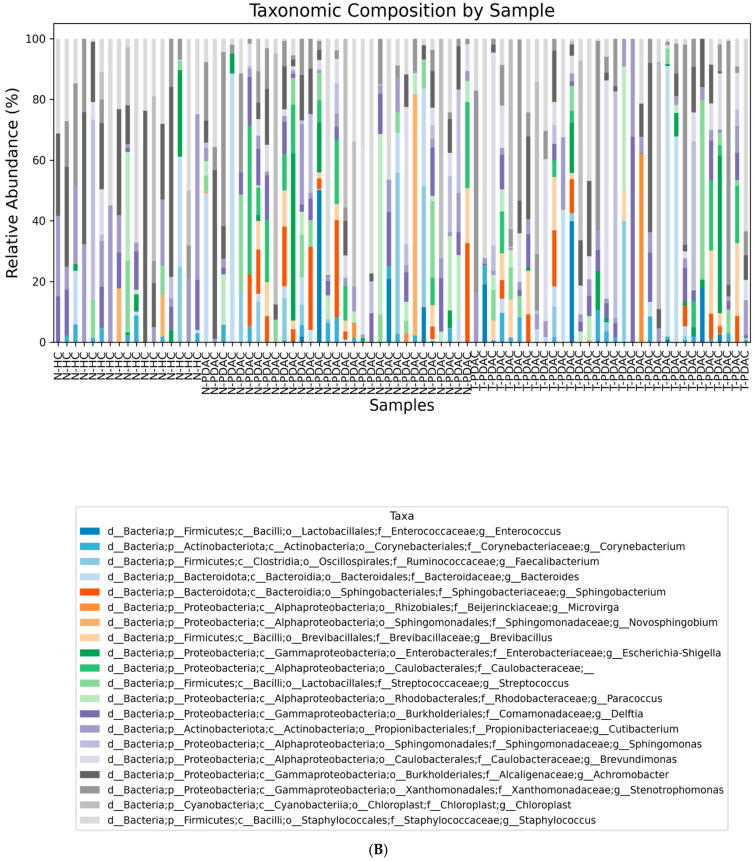
Taxa bar plot. Taxa bar plot representing the relative abundance of the top-20 most abundant bacterial families (**A**) and genera (**B**) within the analyzed samples.

The microbial community compositions across samples can be seen in Figure 1, which displays the 15 most abundant families and genera (relative abundance).

We used the QIIME2 ANCOM module to analyze sample microbial compositions of the samples and identify significant variations among them. After applying a filtering process, feature tables of 29 × 48, 32 × 49, and 34 × 63 genus-collapsed features × samples (see Supplementary Table S1, Sheet S2) were used as input for the ANCOM analysis. The data are shown in Supplementary Figure S3. At the genus level (L6), g_Jeotgalicoccus was found to change significantly between N-HC and N-PDAC (ANCOM W statistics = 27) or T-PDAC (ANCOM W statistics = 25) and N-HC. When T-PDAC were compared to matched N-PDAC tissue specimens, no significant differences were found at either the genus or the species level.

### 3.5. Pairwise Differential Abundance Analysis

The variation in microbial abundance between pairs of groups was further explored using the Coda-lasso, Clr-lasso, and Selbal methods, and we focused on “genus-collapsed” taxa that were found in common among the three methods. The full results are given in Supplementary Table S1 (Sheet S3).

In tissue samples from both N-PDAC and T-PDAC compared to N-HC, two “genus-collapsed” taxa were observed as showing greater abundance in N-HC: *g_Jeotgalicoccus* (N-PDAC vs. N-HC: OTU139; T-PDAC vs. N-HC: OTU114), and a species of *Acinetobacter genus* named *s_guillouiae* (N-PDAC vs. N-HC: OTU2445; T-PDAC vs. N-HC: OTU2351).

When tissue samples from T-PDAC were compared to matched N-PDAC, four “genus-collapsed” taxa were identified as shared across the three methods. In detail, *g_Methylobacterium-Methylorubrum* (OTU1858) and *g_Delftia* (OTU2125) were present in a greater abundance in T-PDAC specimens; conversely, *g_Sphingomonas* (OTU353) and a species of Streptococcus genus named *s_Streptococcus_salivarius* (OTU1149) were more abundant in N-PDAC.

### 3.6. Lack of Significant Associations Between Microbial Taxa and Clinical Features

No significant correlations were observed between the selected clinical features of PDAC patients (age at diagnosis, jaundice, serum levels of CA 19 9, diabetes, presence of mucinous subtype, tumor stage, resection margin status, cancer treatment with neo-adjuvant or adjuvant chemotherapy, or radiotherapy) and microbial taxa (Supplementary Table S2). After applying the Benjamini–Hochberg correction, none of the microbial taxa showed a Pearson correlation coefficient exceeding 0.7 relative to the clinical features (adjusted *p*-value < 0.05). These findings highlight the absence of robust relationships between microbial community composition and the selected clinical features in this dataset. Possible explanations for this lack of association include biological variability, the limited sample size, and the presence of confounding factors not accounted for in the current analysis.

### 3.7. Metabolic Function Analysis

Data on functional metagenomic profiles and pathways with varying abundance via the pairwise comparisons of groups are given in Supplementary Table S3. In the comparison between N-PDAC and N-HC a total of 2884 Enzyme Commission numbers (Sheet S4) and 485 metabolic pathways defined by MetaCyc identifiers (Sheet S5) have been inferred using the PICRUSt pipeline. Using the PICRUSt pathway count table, we identified differentially abundant pathways through pairwise group comparisons. Similarly, in the comparison between T-PDAC and N-HC, a total of 2876 Enzyme Commission numbers (Sheet S6) and 485 metabolic pathways (Sheet S7) were identified. In the comparison between N-PDAC and T-PDAC, a total of 2860 Enzyme Commission numbers (Sheet S8) and 485 metabolic pathways (Sheet S9) have also been inferred.

As shown in Sheet S10, a total of seventeen significant differentially abundant pathways were identified using ALDEx2 in the comparison between N-HC and N-PDAC, and five significant differentially abundant pathways between T-PDAC and N-HC have been highlighted. Additionally, a total of 14 significant differentially abundant pathways emerged in the comparison between N-PDAC and T-PDAC.

**Figure 2:**
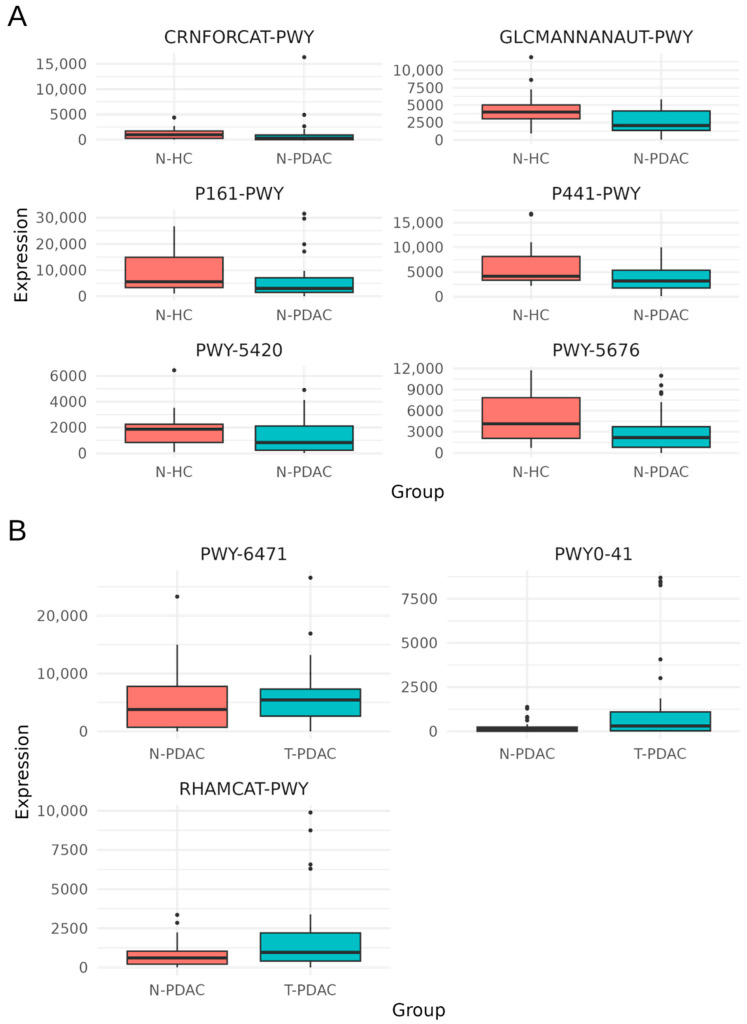
Predicted metabolic functions with higher abundance in specific groups. (**A**) Metabolic functions predicted to have higher abundance in the normal pancreatic tissue from healthy organ donors (N-HC group), compared to the normal tissues from PDAC (N-PDAC group). CRNFORCAT-PWY corresponds to creatinine degradation I; GLCMANNANAUT-PWY corresponds to superpathway of N-acetylglucosamine, N-acetylmannosamine and -acetylneuraminate degradation; P161-PWY corresponds to acetylene degradation (anaerobic); P441-PWY corresponds to superpathway of N-acetylneuraminate degradation; PWY-5420 corresponds to catechol degradation II (meta-cleavage pathway); PWY-5676 corresponds to acetyl-CoA fermentation to butanoate. (**B**) Metabolic functions predicted to have higher abundance in the tumors compared to normal tissues from PDAC (T-PDAC group and N-PDAC group, respectively). PWY-6471 corresponds to peptidoglycan biosynthesis IV (Enterococcus faecium); PWY0-41 corresponds to allantoin degradation IV (anaerobic); RHAMCAT-PWY corresponds to L-rhamnose degradation I.

Significant differentially abundant pathways were filtered by eliminating pathways highlighted in more than one comparison, as well as those that have not been described as to their involvement in human cancer growth or in biological features that may favor its onset and progression. Predicted metabolic functions with higher abundance in specific groups after filtering are shown in Figure 2A,B. The metabolic functions listed below appeared to have a higher predicted abundance in the N-HC group as compared to the N-PDAC one (Figure 2A): creatinine degradation I (MetaCyc identifier: CRNFORCAT-PWY); superpathway of N-acetylglucosamine, N-acetylmannosamine, and N-acetylneuraminate degradation (GLCMANNANAUT-PWY); acetylene degradation (anaerobic) (P161-PWY); superpathway of N-acetylneuraminate degradation (P441-PWY), catechol degradation II (meta-cleavage pathway) (PWY-5420), and acetyl-CoA fermentation to butanoate (PWY-5676). Conversely, no predicted metabolic functions with differential abundance in the N-HC group compared to the T-PDAC group emerged. In addition, when the N-PDAC group was compared to the T-PDAC group (Figure 2B), the following metabolic functions appeared to have a higher predicted abundance in the latter group: allantoin degradation IV (anaerobic) (PWY0-41), peptidoglycan biosynthesis IV (Enterococcus faecium) (PWY-6471), and L-rhamnose degradation I (RHAMCAT-PWY).

## 4. Discussion

Although the pancreas has previously been thought to be a sterile organ, recent studies have highlighted that microbiota exist in PDAC tissue, and tumor-resident bacteria have been suggested to affect anti-tumor immunity and tumor prognosis [36]. The study of intratumoral microbiota in PDAC is an emerging area with significant importance for comprehending pancreatic carcinogenesis and development, prognosis, and treatment strategies. However, the solidity and congruity of findings in this field of research are often defined by various confounding factors, including the challenge of reaching a consensus on the composition of the microbiota and the mechanistic and functional roles of microbes in the pancreatic tissue and normal surrounding tissues of patients with PDAC.

In this investigation, targeted bacterial 16S rRNA gene sequencing was first performed in tumoral and normal tissues from PDAC patients, as compared to normal pancreatic tissues from healthy organ donors, for the purpose of finding microbial features and biological functions enabling researchers to distinguish between subjects affected and unaffected by PDAC; then, the differences in the microbial composition between tumoral and normal tissues from PDAC patients were analyzed to identify the most representative features associated with PDAC development.

### 4.1. Diversity of Microbial Communities

Based on the alpha metrics, we observed a trend towards a lower microbial abundance in tumoral tissue specimens from PDAC compared to those from healthy pancreas donors, while a significantly lower microbial abundance in tumoral specimens from PDAC, compared to matched normal tissues, emerged. On the other hand, all hypothesis-testing provided significant results in terms of beta diversity relative to the tumor and normal specimens from PDAC, compared to those from healthy pancreas donors, while in matched pairs of tumor and normal tissues from PDAC a significant beta diversity emerged only according to the unweighted UniFrac dissimilarity.

### 4.2. Differential Abundance Analysis

By comparing the compositions of microbiomes between the investigated groups, two main subsets of taxa were observed. The first subset was characterized as having a greater abundance in samples from healthy pancreas donors, compared to both normal and tumor tissues from PDAC, and included *g_Jeotgalicoccus* and *s_Acinetobacter_guillouiae*, representing bacteria with a potential beneficial effect and anticancer activity, respectively. Of note, the former emerged from all the compositional data analysis methods used in the bioinformatic pipeline, as well as QIIME2-ANCOM. The second subset was characterized as having different abundances in tumoral compared to normal tissues from PDAC, and encompassed two decreased taxa involved in the establishment of immune homeostasis and in the suppression of tumor progression (*s_Streptococcus_salivarius*, and *g_Sphingomonas*), and two other enhanced taxa with a role in tumor initiation and development (*g_Methylobacterium-Methylorubrum*, and *g_Delftia*).

*Jeotgalicoccus* is a Gram-positive bacteria that can produce butyrate and promote gut homeostasis [37]. To date, no significant associations with PDAC have been reported. However, this microorganism has been associated with late-stage colon, bladder, ovarian, and breast cancers [38,39,40,41].

*Acinetobacter guillouiae* is a nonfermenting, aerobic, Gram-negative bacillus that inhabits the normal flora of the oropharynx, skin, and peritoneum in approximately 25% of all healthy individuals. Crude extracts of this microorganism showed anticancer activity in glioblastoma cell lines; a high bioactive potential against U87MG cell lines by reducing cell growth by 50% has been reported [42]. To our knowledge, the only association between *Acinetobacter guillouiae* and human cancer has been referred to in the context of lung cancer, for which it was among the enriched microbial species in plasma from patients compared to healthy controls [43].

*Streptococcus salivarius* is a Gram-positive, facultative anaerobic microorganism, and pioneer colonizer of the oral cavity and gastrointestinal tract in humans. It is known to promote the establishment of immune homeostasis and management of host inflammatory responses by inhibiting the activation of the nuclear factor kappa B (NF-κβ) pathway [44]. Significant depletion of this taxon in saliva emerged as the most prominent signature in the PDAC oral microbiome, whereas *Streptococcus salivarius* was remarkably more abundant in the fecal microbiota of PDAC patients, compared to control subjects [45,46]. As for the intratumoral microbiota composition, *Streptococcus salivarius* emerged as being highly associated with the tumor site in oral squamous cell carcinoma tumor tissues, compared with adjacent non-tumor mucosa samples [47]. Furthermore, *Streptococcus salivarius* has been among the oral streptococci commonly isolated from cervical lymph nodes in patients with oral cancer [48]. That said, no evidence about the presence of *Streptococcus salivarius* in pancreatic tissues has been reported to date.

*Sphingomonas* are Gram-negative, strictly aerobic bacteria known to have the capacity to activate natural killer T cells, which hinder tumor growth [49]. By performing 16S rRNA amplicon sequencing in tumor samples resected from long-term and short-term PDAC survivors, *Sphingomonas* genera were identified as being associated with long-term survival in PDAC [50]. Furthermore, the evaluation of the microbial composition on bacteria-derived extracellular vesicles acquired from blood samples of PDAC patients and healthy controls showed that *Sphingomonas* was less abundant in PDAC, compared to controls [51]. At the tissue level, a reduction of *Sphingomonas* in hepatocellular carcinoma compared to adjacent non-tumor tissue specimens has been reported [52], whereas elevated abundance of this taxon has been highlighted in gastric cancer, compared to adjacent non-cancerous tissues [53], in deep tissue compared to tumor surfaces in oral cancer [54], and in thymic epithelial tumors [55].

*Methylobacterium-Methylorubrum* is a pink-pigmented, aerobic, facultatively methylotrophic Gram-negative bacterium. It was found to be significantly increased in tissue from distal gastric cancers, positively correlated with cancer-promoting metabolites, and negatively correlated with cancer-inhibiting metabolites [56]. Moreover, the intratumoral *Methylobacterium* was significantly associated with poor prognosis in patients with gastric cancer; the high abundance of *Methylobacterium* was inversely correlated with the frequency of CD8+ tissue-resident memory T cells in the tumor microenvironment and the TGFβ expression, thus playing a role in gastric carcinogenesis [57]. *Methylobacterium-Methylorubrum* was also specific to the endometrial microbiota in cancerous tissues [58]. One study, aiming to investigate a potential intracystic pancreatic microbiome in a pancreatic cystic neoplasm surgery patient cohort, found that *Methylobacterium* was more abundant in cystic fluid and plasma from IPMN with low-grade dysplasia and invasive IPMN [59].

As reported, the Gram-negative bacterium *Delftia* causes different types of infections in immunocompromised patients, including cancer [60,61], suggesting its contribution to risk stratification for pancreatic cancer. A previous study performed comparative profiling of bacterial populations in pancreatic tumors and their respective adjacent normal tissues and revealed an enrichment of *Delftia* in tumor tissues. Furthermore, the level of bacterial genus *Delftia* was higher in well-differentiated compared with moderately and poorly differentiated PDAC, suggesting its roles in tumor initiation and development, and in relation to the characteristics of well-differentiated cells. The presence of this taxon was positively correlated with increased expression of immune checkpoint-associated proteins, such as PD-L1 [62]. Other evidence on the enrichment of *Delftia* in tissue from cancer patients exists, albeit with associations opposite to those for cancer tissue, in patients with hepatocellular carcinoma and colorectal cancer [63,64].

### 4.3. Prediction of Metagenome Functional Content

What stands out from our analysis of the metabolic functions obtained from the microbial gene content inferred from the 16S rRNA gene data is that, when compared to samples from normal pancreatic tissue from PDAC, the samples from healthy pancreas donors showed enrichment of functions involved in fatty acid synthesis (acetylene degradation; acetyl-CoA fermentation to butanoate). As acetylene can be metabolized to acetyl-CoA and then to acetate and butyrate; the achieved results point to a decreased production of short-chain fatty acids (SCFAs) in PDAC. To date, SCFAs are among the most studied microbiota-derived factors in the development, progression, and clinical outcomes of PDAC [65]. The relative abundance of butyrate-producing bacteria in the gut of PDAC patients was found to be decreased, and lower concentrations of butyrate in fecal samples of PDAC patients were also reported [66,67]. Furthermore, butyrate has been associated with improved prognosis and enhanced response to chemotherapy in PDAC in in vitro and in vivo models. Noteworthy herein, in line with data reported in the literature [51], is that the genus Actinobacteria, which is known to produce butyrate and modulate immune function, was less abundant in PDAC patients compared to healthy pancreas donors. Although this has to be thoroughly validated in future studies, it could be hypothesized that the lower levels of SCFAs, and mainly of butyrate, in PDAC patients may contribute to the modulation of several processes involved in PDAC development.

The analysis of the inferred metabolic functions in the tumoral compared to normal tissues from PDAC identified the peptidoglycan biosynthesis IV (Enterococcus faecium) and the L-rhamnose degradation as being among the functions more abundant in tumor tissues. The enhancement of peptidoglycan biosynthesis has been reported in a previous study aiming to explore the changes in gut microbiota in the progression of PDAC [68]. In addition, bacterial pathway analysis identified the over-representation of this pathway in the oral microbiome of non-smoker patients with oral-cavity squamous cell carcinoma [69], and a switch towards peptidoglycan biosynthesis has also been observed in gastric cancer [70,71,72]. This data suggests a role for peptidoglycan biosynthesis in PDAC development, as supported by previous observations as to other cancers.

As for the L-rhamnose degradation function, although much more research is required to elucidate the mechanism of action of this 6-deoxy methyl pentose sugar, it has been hypothesized that rhamnose could exert its anticancer effect via negatively influencing fucosylation, and thus the behavior of malignant cells [73]. To our knowledge, no evidence exists on L-rhamnose degradation in PDAC. However, this function emerged as being associated with shorter progression-free survival in patients with melanoma undergoing immunotherapy [74].

## 5. Weaknesses and Strengths, Conclusions and Future Perspectives

We are aware that this study has several limitations. First, the number of patients included in the present investigation, although this is to be considered in light of the single-center nature of the study, and the shortage of sufficient power to examine the possible correlations between the intratumoral bacterial composition and clinical pathological features up to clinical outcomes in PDAC patients, did not allow researchers to reach broad conclusions. Furthermore, the patients in our cohort consisted solely of individuals with resectable disease, which may limit the relevancy of the presented findings in patients with advanced metastatic disease. Second, we recognize the lack of adjustment for potential existing environmental and lifestyle-related factors such as geographical location, BMI, smoking, medication (including antibiotics), and dietary habits, and for confounding factors including the precautions made to reduce contamination during collection and processing of tissue samples. We are also aware that the lack of demographic data for the heathy organ donors made it difficult to evaluate potential age- and gender-related differences in the microbiota in the control group. Third, we know that a “shotgun metagenomics” approach would have allowed the achievement of a higher taxonomic coverage and resolution, and above all, allowed researchers to profile microbial genes, allowing for more powerful identification of microbial function, with the potential to differentiate between “healthy” and “diseased” microbiomes in PDAC. However, we considered that tissue samples display a heightened human-host background compared to other specimens, such as feces, which leads to a lower quantity and ratio of microbial reads, thereby diminishing the sensitivity of shotgun metagenomic sequencing [75]. Therefore, within the limits of the available resources, 16S amplicons sequencing and bioinformatic tools to predict microbiome function with 16S rRNA gene data were applied in our cohort.

The real strength of this study is undoubtedly represented by the bioinformatics data analysis pipeline. Indeed, multiple rarefaction tests were applied to identify the optimal coverage threshold for the majority of samples, ensuring robustness and accuracy in microbial diversity assessments. Additionally, a variety of computational methods were used to analyze group differences, enhancing the reliability of the findings. The integration of these diverse approaches enabled a robust prediction of microbial functions, with data shared across the methods, reinforcing the consistency of the results.

Overall, in the attempt to determine the intratumoral microbiota signature in PDAC net ofthe microbiota from non-cancer subjects, our findings underscored the microbial diversity levels in tissues from PDAC compared to those from healthy pancreas donors, with decreased abundances of bacteria with potential beneficial effect and anticancer activity found in PDAC. Moreover, significant differences emerged between matched tumoral and normal tissues in PDAC, with the reduction of bacteria and microbial functions involved in the establishment of immune homeostasis and in the suppression of tumor progression, and the enhancement of those with a potential role in tumor initiation and development. In light of these data, it may be that our methodological approach could provide a roadmap for future studies aimed at dissecting the microbiota associated with PDAC development.

Future prospective studies focusing on larger patient populations should incorporate potential existing confounding factors such as environmental elements and lifestyle-related habits, and should also include, besides resectable PDAC, patients in advanced tumor stages, making it possible to draw more generalizable conclusions about intratumoral bacteria alterations in PDAC. Moreover, the associations underscored in this study require further functional investigations to prove and clarify the correlations and causality between microbiota and pathogenicity in PDAC.

## Figures and Tables

**Table 1:** Demographics, clinical presentation, anamnestic information, and clinical pathological features of 32 patients with pancreatic ductal adenocarcinoma (PDAC) retrospectively enrolled into the study.

Gender, N (%)	
Male	21 (65.6)
Female	11 (34.4)
Age, median (IQR)	59.5 (51–73)
<55 years, N (%)	13 (40.6)
≥55 years, N (%)	19 (59.4)
Obstructive jaundice, yes/no (%yes)	11/20 (35.5)
missing, N (%)	1 (3.1)
Diabetes, yes/no (%)	9/23 (28.1)
CA 19 9 ≥ 100 U/mL, yes/no (%yes)	14/15 (48.3)
missing, N (%)	2 (6.3)
Personal history of cancer, yes/no (%yes)	2/30 (6.3)
Family history of cancer, yes/no (%yes)	3/29 (9.4)
Tumor location, N (%)	
Head	30 (93.75)
Body/Tail	2 (6.25)
Mucinous PDAC, yes/no (%yes)	6/26 (18.8)
Tumor grade, N (%)	
G1: well-differentiated	4 (12.9)
G2: moderately differentiated	19 (61.3)
G3: poorly differentiated	8 (25.8)
missing, N (%)	1 (3.1)
Tumor stage, N (%)	
IB	4 (13)
IIA	1 (3)
IIB	10 (31)
III	16 (50)
IV	1 (3)
Vascular invasion, yes/no (%yes)	4/28 (13)
Perineural invasion, yes/no (%yes)	16/16 (50)
Resection margins, positive/negative (%yes)	6/26 (18.8)
Neo-adjuvant therapy, yes/no (%yes)	2/30 (6.3)
Adjuvant therapy, yes/no (%yes)	22/7 (75.9)
missing, N (%)	3 (9.4)

**Table 2:** Summary of pairwise comparisons among groups for various alpha-diversity indices. Benjamini–Hochberg adjusted *p*-values (q-values) for Kruskal–Wallis tests are presented.

	N-HC vs. N-PDAC	N-HC vs. T-PDAC	T-PDAC vs. N-PDAC
Pielou’s Evenness	0.644	0.644	0.644
Number of Observed Features	0.948	0.322	0.211
Shannon’s Entropy	0.690	0.455	0.424
Simpson Index	0.974	0.619	0.342
Faith’s Phylogenetic Distance	0.383	0.08	0.003

**Table 3:** Summary of pairwise comparisons between groups for beta-diversity metrics. The corrected *p*-values (q-values) for PERMANOVA tests by Benjamini–Hochberg are presented.

	N-HC vs. N-PDAC	N-HC vs. T-PDAC	T-PDAC vs. N-PDAC
Bray–Curtis dissimilarity	0.002	0.002	0.845
Jaccard distance	0.002	0.002	0.307
Weighted UniFrac dissimilarity	0.042	0.012	0.602
Unweighted UniFrac dissimilarity	0.009	0.009	0.024

## Data Availability

The raw sequence data have been deposited in the National Center for Biotechnology Information (NCBI) under the BioProject ID PRJNA1222158 and can be accessed at https://www.ncbi.nlm.nih.gov/bioproject/1222158.

## Supplementary Materials

The following supporting information can be downloaded at: https://www.mdpi.com/article/10.3390/microorganisms13020452/s1, Supplementary Table S1, Sequencing and alignment statistics and composition analyses. Sheet S1, Sequencing and alignment statistics, and feature distribution per sample (“per sample” column) and global feature distribution (“per feature” column). Sheet S2, Genus-collapsed feature table from QIIME2. Column A: feature custom identifier; column B: full taxonomic classification; columns C-BR: feature counts per sample. Sheet S3, Taxonomic Features Identified by Variable Selection Methods. Supplementary Table S2, Pearson correlation coefficients between microbial taxa and clinical features. Supplementary Table S3, Functional metagenomic profiles. Sheet S4, Enzyme Commission (EC) Gene Function Abundance. This table presents the relative abundance of gene functions defined by Enzyme Commission (EC) identifiers across the samples. The data are based on pairwise group comparisons between N-HC and N-PDAC. Sheet S5, MetaCyc Pathway Abundance. This table displays the relative abundance of metabolic pathways defined by MetaCyc identifiers across the samples. The data are based on pairwise group comparisons between N-HC and N-PDAC. Sheet S6, Enzyme Commission (EC) Gene Function Abundance. This table presents the relative abundance of gene functions defined by Enzyme Commission (EC) identifiers across the samples. The data are based on pairwise group comparisons between N-HC and T-PDAC. Sheet S7, MetaCyc Pathway Abundance. This table displays the relative abundance of metabolic pathways defined by MetaCyc identifiers across the samples. The data are based on pairwise group comparisons between N-HC and T-PDAC. Sheet S8, Enzyme Commission (EC) Gene Function Abundance. This table presents the relative abundance of gene functions defined by Enzyme Commission (EC) identifiers across the samples. The data are based on pairwise group comparisons between N-PDAC and T-PDAC. Sheet S9, MetaCyc Pathway Abundance. This table displays the relative abundance of metabolic pathways defined by MetaCyc identifiers across the samples. The data are based on pairwise group comparisons between N-PDAC and T-PDAC. Sheet S10, Differentially Abundant Pathways. This table lists the metabolic pathways identified as differentially abundant between groups, as determined by ALDEx2. Supplementary Figure S1, Alpha-diversity analysis. We investigated Alpha diversity by calculating five different measures: Pielou’s evenness, Number of Observed Features, Shannon’s Entropy, Simpson Index, and Faith’s Phylogenetic Distance. For each index, the following outcomes are provided: boxplot for group-specific index distribution (*x*-axis: “N_HC”, “T_PDAC”, and “N_PDAC”; *y*-axis: alpha-diversity index); Wallis test results for pairwise group comparisons. Analyses were performed through the “QIIME2 Diversity” module, using “8573” as cut-off for reads sampling depth (see main text for further details). Supplementary Figure S2, Beta-diversity analysis. We investigated Beta-diversity between the two groups by evaluating four dissimilarity/distance methods: Bray-Curtis, Jaccard, and unweighted and weighted Unifrac. For each method, a screenshot of three-dimensional Principal Coordinate Analysis (“3D-PCoA”) plot, obtained by using the Emperor web-application within QIIME2 website (https://view.qiime2.org/), and a table summarizing PERMANOVA test results for pairwise group comparisons are provided. YELLOW dots indicate healthy pancreatic tissue samples from organ donors “N-HC”, BLUE dots indicate pancreatic tissue non-tumor specimens collected from PDAC cases “N-PDAC”, and RED dots indicate pancreatic tissue tumor specimens collected from PDAC cases “T-PDAC”. Each table shows details comparing groups and their sizes, number of permutations, pseudo-F statistics, *p*-values and Benjamini–Hochberg adjusted *p*-value. PERMANOVA verifies the hypothesis that distances among samples with one class group differ from distances of these samples from samples of other groups. Supplementary Figure S3, ANCOM. A QIIME2 ANCOM module was used to infer microbial genera or species that are differentially abundant across sample groups. Rare features and mitochondrial/plastid sequences were removed from the feature table (see Section 2). Thus, features were collapsed according to QIIME2 taxonomical classification (based on the GreenGenes resource) into “genus-collapsed” (“level 6”) features, for totals of 29, 32, and 34 taxa, respectively (see Supplementary Table S2). ANCOM volcano plot and statistical results table are provided. Significant features are placed on the top-right corner of the volcano plot; the first table shows significant features, together with the corresponding W statistics, i.e., the number of sub-hypotheses that have passed for a certain feature (ANCOM compares pairs of feature-relative abundances). The second table shows the percentile abundances of relevant features across the sample groups. For example, a value of 100 at the 50% percentile indicates that the detected feature has a maximum sequence count of 100 in 50% of samples of an investigated group.
