# Supplementary material for: Could the Microbial Profiling of Normal Pancreatic Tissue from Healthy Organ Donors Contribute to Understanding the Intratumoral Microbiota Signature in Pancreatic Ductal Adenocarcinoma?
Source: Microorganisms. 2025 Feb 19;13(2):452. doi: 10.3390/microorganisms13020452 (PMC11858623; doi:10.3390/microorganisms13020452)

**Supplementary Figure S1. Alpha diversity analysis.**

We investigated Alpha diversity by calculating five different measures: Pielou's evenness, Number of Observed Features. and Shannon's entropy, Simpson, and Faith's Phylogenetic Distance. For each index, the following outcomes are provided: boxplot for group-specific index distribution (x-axis: "N_HC", "T_PDAC", and "N_PDAC"; y-axis: alpha-diversity index); -Wallis test results for pairwise group comparisons. Analyses were performed through the "QIIME2 Diversity" module, using "8,573" as cut-off for reads sampling depth (see main text for further details).

<u>**Pielou's evenness**</u>

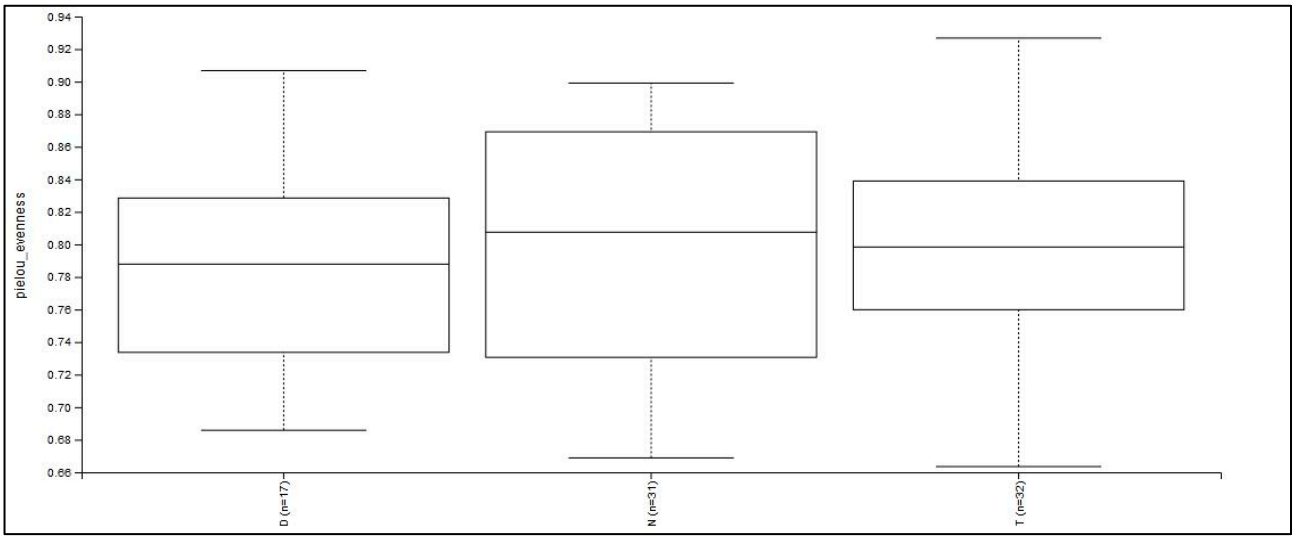


Kruskal-Wallis test (pairwise):

| Group 1 | Group 2 | H | p-value | q-value |
|---|---|---|---|---|
| N-HC (n=17) | N-PDAC (n=31) | 0.432 | 0.511 | 0.644 |
| | T-PDAC (n=32) | 0.214 | 0.644 | 0.644 |
| N-PDAC (n=31) | T-PDAC (n=32) | 0.333 | 0.564 | 0.644 |

<u>**Number of observed Features**</u>

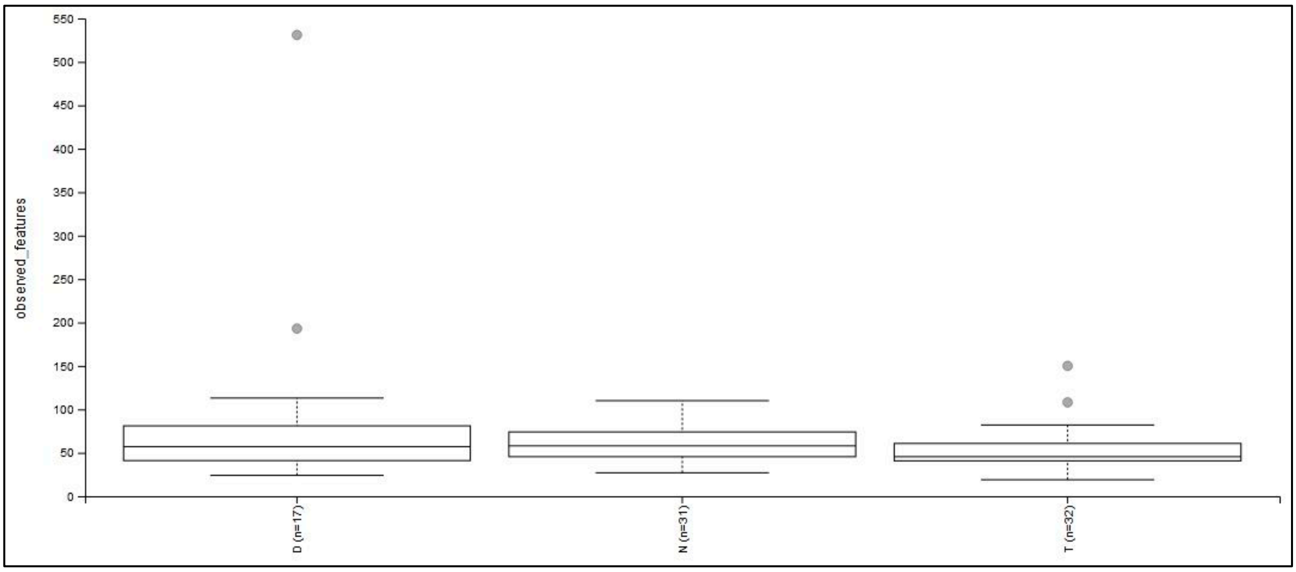

Kruskal-Wallis test (pairwise):

| Group 1 | Group 2 | H | p-value | q-value |
|---|---|---|---|---|
| N-HC (n=17) | N-PDAC (n=31) | 0.004 | 0.948 | 0.948 |
| | T-PDAC (n=32) | 1.539 | 0.215 | 0.322 |
| N-PDAC (n=31) | T-PDAC (n=32) | 3.273 | 0.070 | 0.211 |

**Shannon's entropy**

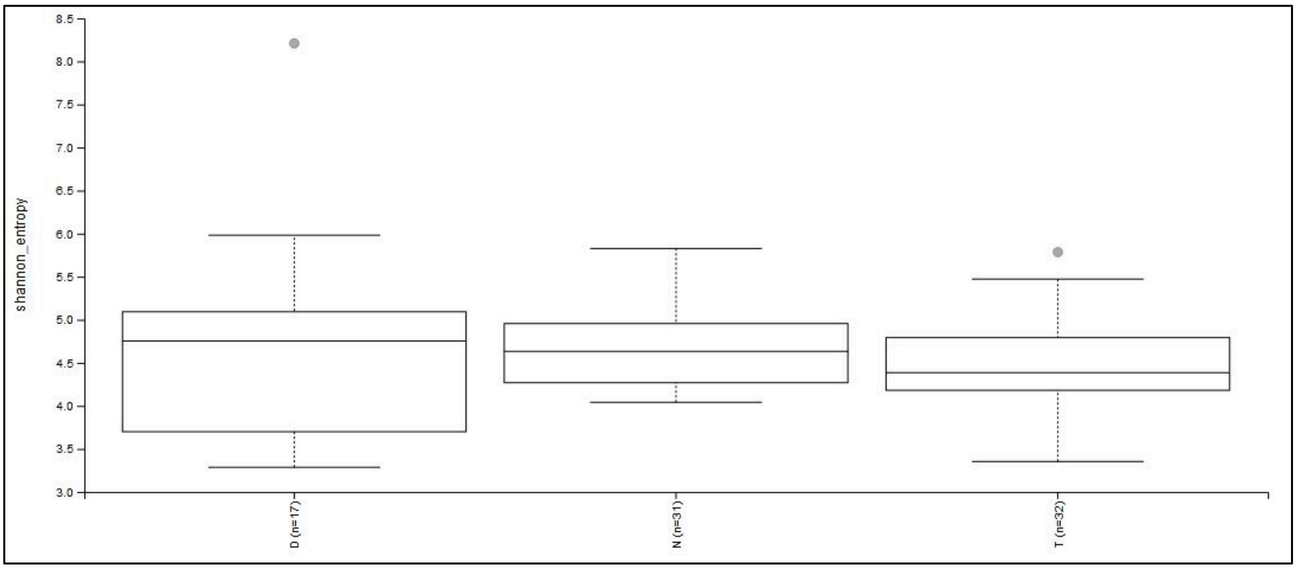


Kruskal-Wallis test (pairwise):

| Group 1 | Group 2 | H | p-value | q-value |
|---|---|---|---|---|
| N-HC (n=17) | N-PDAC (n=31) | 0.159 | 0.690 | 0.690 |
| | T-PDAC (n=32) | 1.059 | 0.303 | 0.455 |
| N-PDAC (n=31) | T-PDAC (n=32) | 2.164 | 0.141 | 0.424 |

**Simpson**

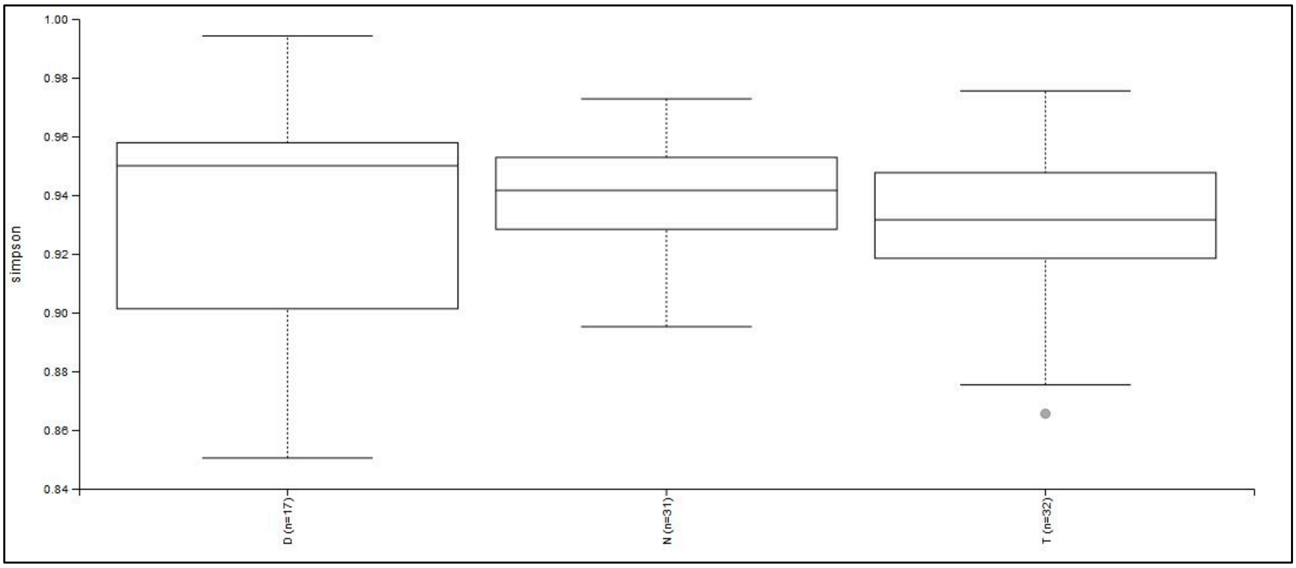

Kruskal-Wallis test (pairwise):

| Group 1 | Group 2 | H | p-value | q-value |
|---|---|---|---|---|
| N-HC (n=17) | N-PDAC (n=31) | 0.001 | 0.974 | 0.974 |
| | T-PDAC (n=32) | 0.671 | 0.413 | 0.619 |
| N-PDAC (n=31) | T-PDAC (n=32) | 2.500 | 0.114 | 0.342 |

**Faith's Phylogenetic Distance**

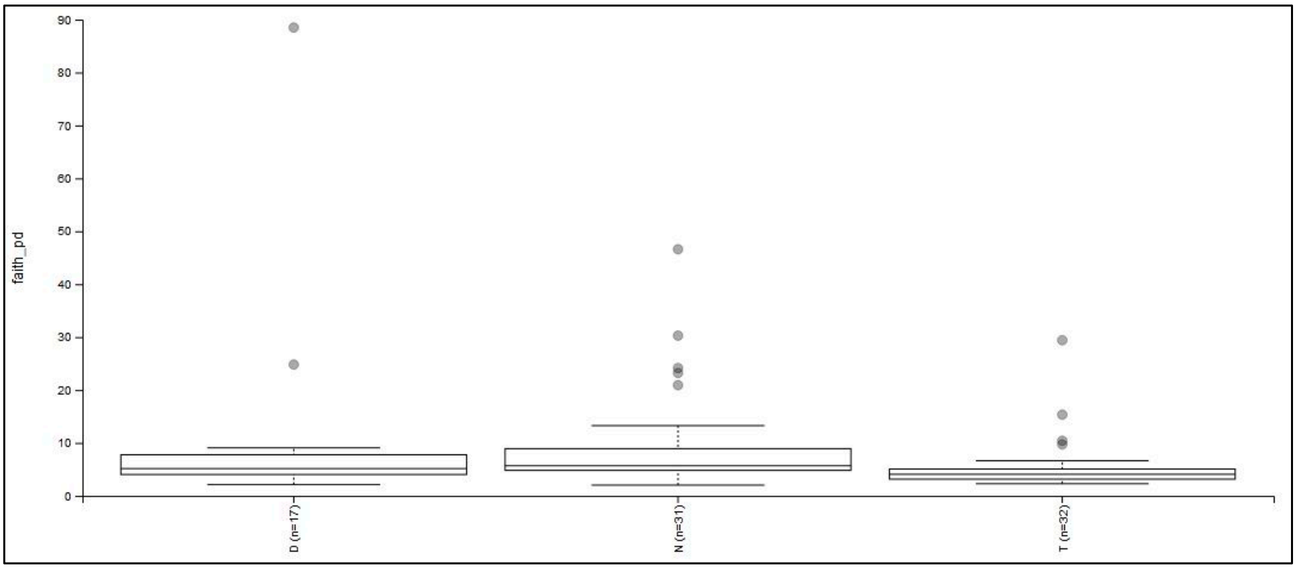


Kruskal-Wallis test (pairwise):

| Group 1 | Group 2 | H | p-value | q-value |
|---|---|---|---|---|
| N-HC (n=17) | N-PDAC (n=31) | 0.762 | 0.383 | 0.383 |
| | T-PDAC (n=32) | 3.734 | 0.053 | 0.080 |
| N-PDAC (n=31) | T-PDAC (n=32) | 10.617 | 0.001 | 0.003 |

**Supplementary Figure S2_ Beta diversity analysis.**

We investigated Beta diversity between the two groups by evaluating four dissimilarity/distance methods: Bray-Curtis, Jaccard, Unweighted and Weighted Unifrac. For each method, a screenshot of three-dimensional Principal Coordinate Analysis ("3D-PCoA") plot, obtained by using the Emperor web-application within QIIME2 website (https://view.qiime2.org/), and a table summarizing PERMANOVA test results for pairwise group comparisons are provided. YELLOW dots indicate healthy pancreatic tissue samples from organ donors "N-HC", BLUE dots indicate pancreatic tissue non-tumor specimens collected from PDAC cases "N-PDAC", and RED dots indicate pancreatic tissue tumor specimens collected from PDAC cases "T-PDAC". In details, each table show details on comparing groups and their size, number of permutations, pseudo-F statistics, p-values and Benjamini-Hochberg adjusted p-value. PERMANOVA verifies the hypothesis that distances among samples with one class group differ from distances of these samples from samples of other groups.

**Bray-Curtis**

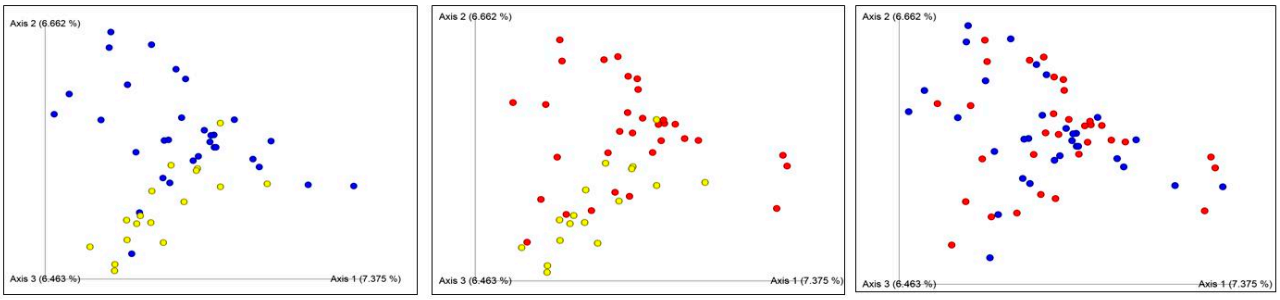


| Group 1 | Group 2 | Sample size | Permutations | pseudo-F | p-value | q-value |
|---|---|---|---|---|---|---|
| N-HC | N-PDAC | 48 | 999 | 2.570 | 0.001 | 0.002 |
| | T-PDAC | 49 | 999 | 2.760 | 0.001 | 0.002 |
| N-PDAC | T-PDAC | 63 | 999 | 0.778 | 0.845 | 0.845 |

**Jaccard**

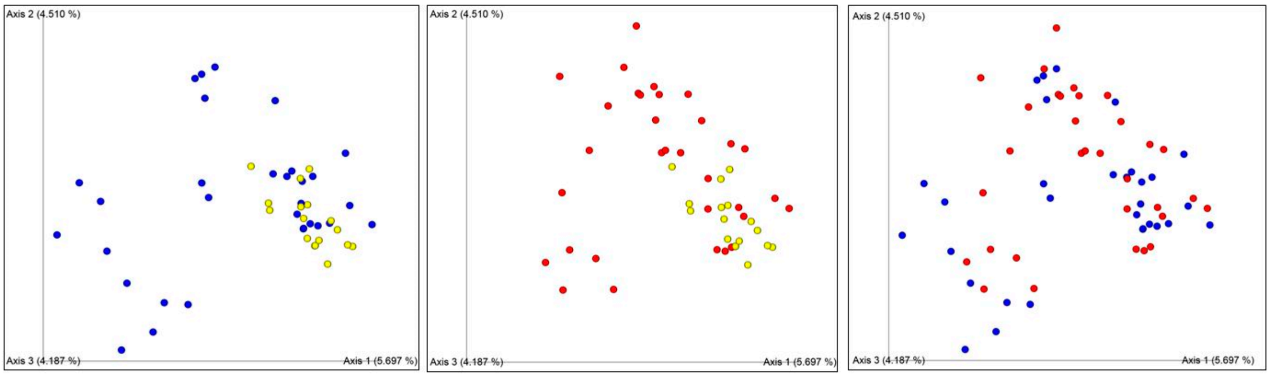


| Group 1 | Group 2 | Sample size | Permutations | pseudo-F | p-value | q-value |
|---|---|---|---|---|---|---|
| N-HC | N-PDAC | 48 | 999 | 2.258 | 0.001 | 0.002 |
| | T-PDAC | 49 | 999 | 2.432 | 0.001 | 0.002 |
| N-PDAC | T-PDAC | 63 | 999 | 1.057 | 0.307 | 0.307 |

**Weighted Unif**

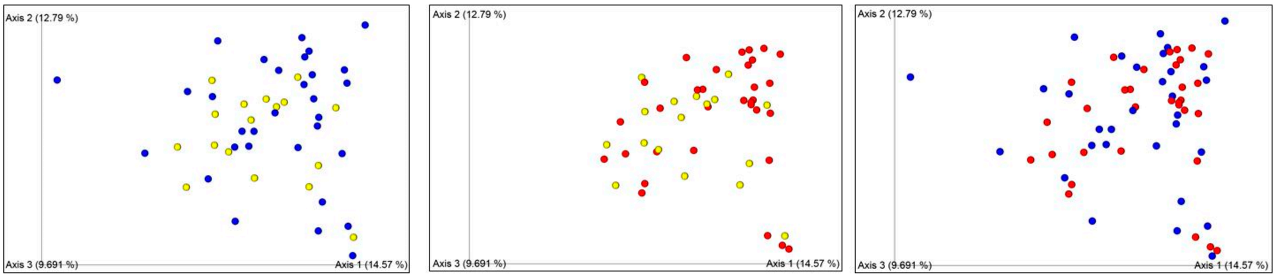


| Group 1 | Group 2 | Sample size | Permutations | pseudo-F | p-value | q-value |
|---|---|---|---|---|---|---|
| N-HC | N-PDAC | 48 | 999 | 1.847 | 0.028 | 0.042 |
| | T-PDAC | 49 | 999 | 2.584 | 0.004 | 0.012 |
| N-PDAC | T-PDAC | 63 | 999 | 0.870 | 0.602 | 0.602 |

**Unweighted Unif**

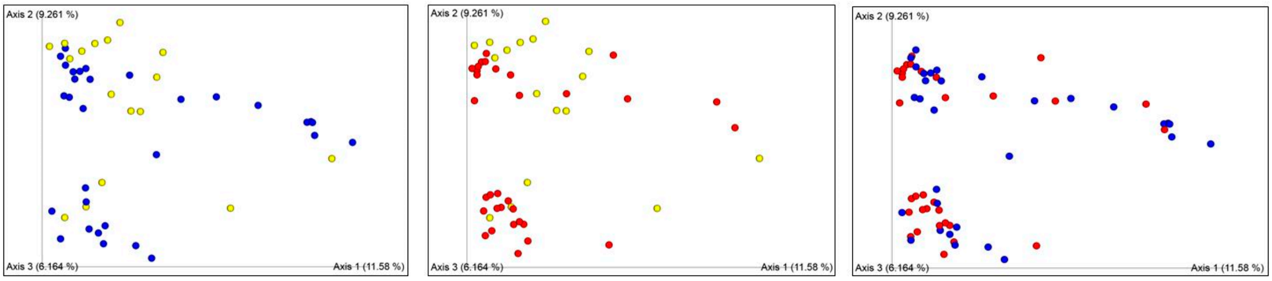


| Group 1 | Group 2 | Sample size | Permutations | pseudo-F | p-value | q-value |
|---|---|---|---|---|---|---|
| N-HC | N-PDAC | 48 | 999 | 1.869 | 0.006 | 0.009 |
| | T-PDAC | 49 | 999 | 2.012 | 0.004 | 0.009 |
| N-PDAC | T-PDAC | 63 | 999 | 1.553 | 0.024 | 0.024 |

**Supplementary Figure S3_ANCOM**

QIIME2 ANCOM module was used to infer microbial genera or species that are differentially abundant across sample groups. Rare features and mitochondrial/plastid sequences were removed from the feature table (see "Materials and methods" section). Thus, features were collapsed according to QIIME2 taxonomical classification (based on GreenGenes resource) into "genus-collapsed" ("level 6") features, for a total of 29, 32 and 34 taxa, respectively (see Supplementary Table 2). ANCOM Volcano Plot and statistical results table are provided.

Significant features are placed on the top-right corner of the Volcano Plot; the first table shows significant features together with the corresponding W statistics, i.e., the number of sub-hypotheses that have passed for a certain feature (ANCOM compares pairs of feature relative abundances). The second table shows the percentile abundance of relevant features across the sample groups. For example, a value of 100 at 50% percentile indicates that the detected feature has a maximum sequence count of 100 in the 50% of samples of an investigated group.

## N-HC vs N-PDAC: Results for genus-collapsed features

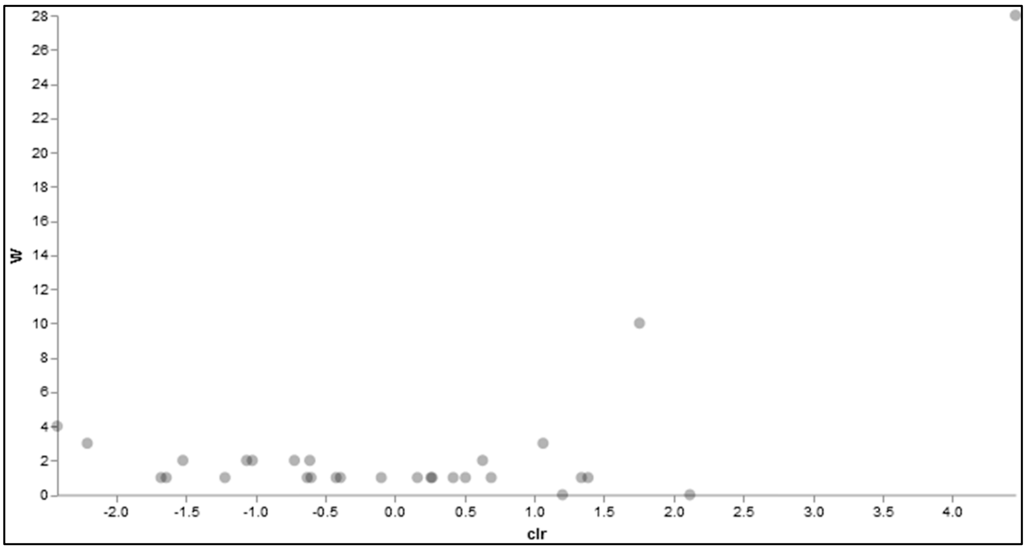


| Feature | W |
|---|---|
| *k__Bacteria;p__Firmicutes;c__Bacilli;o__Bacillales;f__Staphylococcaceae;g__Jeotgalicoccus* | 28 |

| Feature | Percentile | N-HC | N-PDAC |
|---|---|---|---|
| *k__Bacteria;p__Firmicutes; c__Bacilli;o__Bacillales;* | 0.0 | 1.0 | 1.0 |
| *f__Staphylococcaceae;* | 25.0 | 1.0 | 1.0 |
| *g__Jeotgalicoccus* | 50.0 | 300.0 | 1.0 |
| | 75.0 | 925.0 | 1.0 |
| | 100.0 | 2520.0 | 1.0 |

## N-HC vs T-PDAC: Results for genus-collapsed features

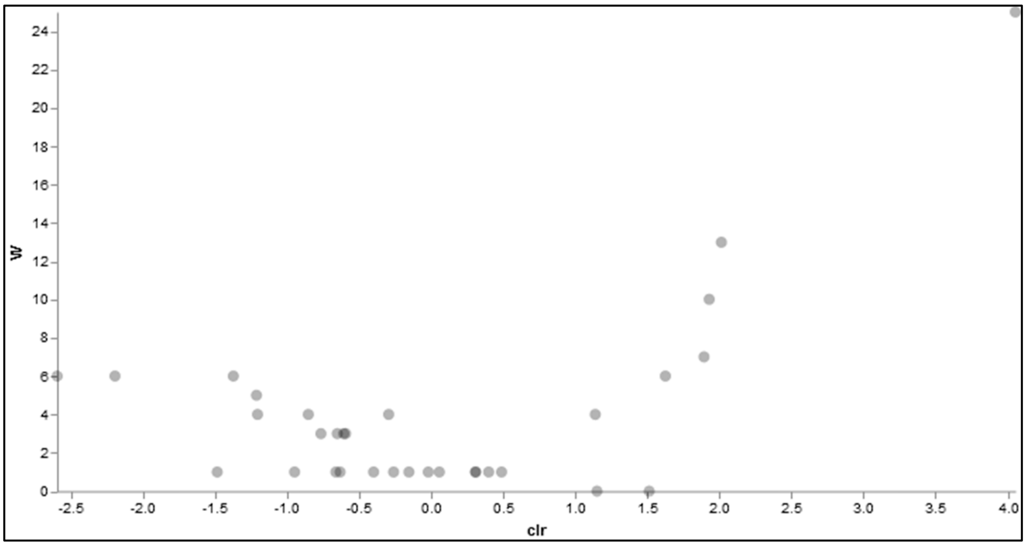


| Feature | W |
|---|---|
| *k__Bacteria;p__Firmicutes;c__Bacilli;o__Bacillales;f__Staphylococcaceae;g__Jeotgalicoccus* | 25 |

| Feature | Percentile | N-HC | T-PDAC |
|---|---|---|---|
| *k__Bacteria;p__Firmicutes; c__Bacilli;o__Bacillales; f__Staphylococcaceae; g__Jeotgalicoccus* | 0.0 | 1.0 | 1.0 |
| | 25.0 | 1.0 | 1.0 |
| | 50.0 | 300.0 | 1.0 |
| | 75.0 | 925.0 | 1.0 |
| | 100.0 | 2520.0 | 658.0 |

**N-PDAC vs T-PDAC: Results for genus-collapsed features**

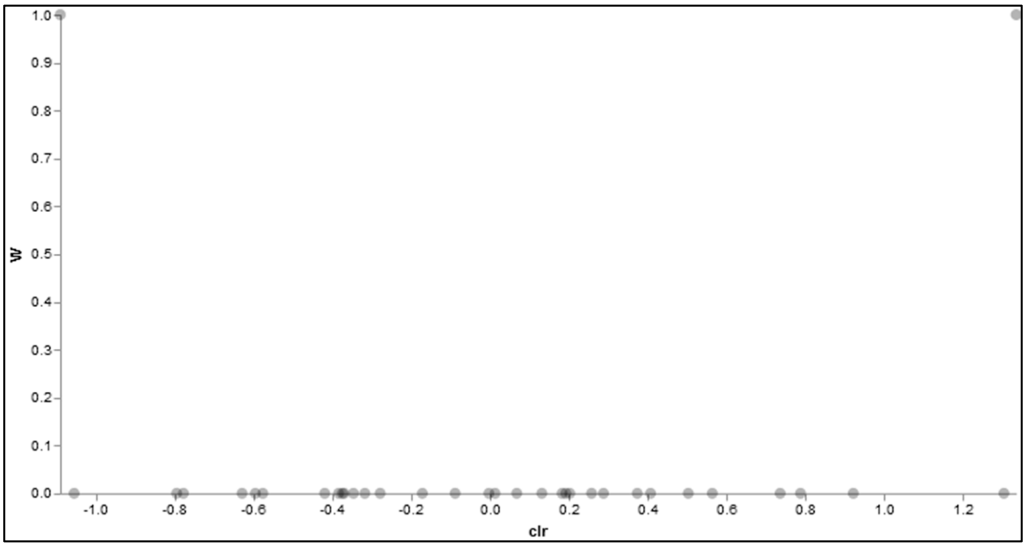

Supplement: Supplementary file 1 [file microorganisms-13-00452-s001.zip › Supplementary files/Supplementary Figures.pdf]
